# The influence of emotional intelligence on academic stress among medical students in Neyshabur, Iran

**DOI:** 10.1186/s12888-023-05344-0

**Published:** 2023-11-16

**Authors:** Elham Charoghchian Khorasani, Mohammad Ardameh, Seyedeh Belin Tavakoly Sany, Hadi Tehrani, Vahid Ghavami, Mahdi Gholian-aval

**Affiliations:** 1 Social Determinants of Health Research Center, Mashhad University of Medical Sciences, Mashhad, Iran; 2 Department of Health Education and Health Promotion, School of Health, Mashhad University of Medical Sciences, Mashhad, Iran; 3 Department of Health, Safety and Environment, Faculty of Health, Mashhad University of Medical Sciences, Mashhad, Iran; 4 Department of Health Education and Health Promotion, Mashhad University of Medical Sciences, Mashhad, Iran; 5 Department of Biostatistics, School of Health, Mashhad University of Medical Sciences, Mashhad, Iran

**Keywords:** Academic stress, Emotional intelligence, Mental health, Students, Health education, Health promotion

## Abstract

**Background:**

Although, several novel strategies related to coping with stress dominate the possible vicissitudes that may occur, academic stress and its mental and physical outcomes remain a serious public concern among college students. Available data on how/where intervention-based novel strategies and emotional intelligence skills can influence students' ability to deal with stress and crisis situations is still unclear. This study aims to investigate the effectiveness of an educational intervention based on emotional intelligence on the level of academic stress components among Iranian medical students.

**Methods:**

This research has been done in two descriptive and quasi-experimental sections in the academic year 2018–2019. To determine the effect of emotional intelligence components on stress levels, this descriptive study was performed on 200 students. Then, a quasi-experimental study was then conducted to determine the impact of an emotional intelligence component-based educational intervention on academic stress-coping skills. Data were collected through a personal information questionnaire, Bradbury and Graves's emotional intelligence questionnaire, and Gadzella’s academic stress questionnaire.

**Results:**

Most of the participants were female (72.3%) married (72%), non-native (62.1%), and second or third academic years (78.5%). The mean number of academic years of employment was 9.5. The mean age of students were 23 ± 3.5 years old. Intervention based on emotional intelligence significantly (*p* < 0.05) improved students’ emotional intelligence skills and decreased their academic stress and reactions to stressors in the intervention group.

**Conclusion:**

It appears that emotional intelligence training is a feasible and highly acceptable way to develop coping skills with academic stress; therefore, such training is essential to be considered as part of university education to improve students’ education quality and their skills to study without academic stress.

## Introduction

The academic transition to university is a time of many changes in students’ lifestyles. University students often experience high academic stress and anxiety due to the peak of workloads, and they coincide with the adaptation to new colleagues and teachers, learning new content, incorporation into the labor market, separation process from the family, curricular reorganizations, and selective and demanding assessments [1]. This causes a series of challenges and overwhelming emotional burdens in the habits of university students, which can negatively affect students’ academic activities. This continuous pressure may lead to translates into different problems and the exhaustion of the student’s reserves; therefore, academic stress appears [2, 3].

Stress is present throughout human life, but it reaches its peak during the academic period of university, and if the student fails to respond appropriately, academic stress develops [4]. Academic stress is one of the most common types of stress and is defined as a “psychological state of the individual generated by continuous social and personal pressure that produces a depletion of the individual’s reserves”. This stress could be managed by arising a person's awareness, perception, and attitude about what need to do and work through each challenge [5]. Academic stress is due to the tendency of today's world to get the most information in the shortest time, in a competitive environment where the minds of people with this type of stress are not able to quickly analyze the mass of information they face, and this leads to physical injuries, become psychological and social [2, 3]. Several studies in the educational field reported that stress is positively associated with depression and anxiety [6], physical illness [7], behavioral disorders [8], mental disorders [9], academic problems [10], and dropping school [11, 12]. However, there are essential mechanisms related to adaptive habits to dominate the possible vicissitudes that may occur. In the last two decades, the concept of emotional intelligence has developed, which can answer many problems not only in psychological and theoretical challenges but also in management problems related to education and public health aspects [13].

Emotional intelligence is a relatively new term that refers to a set of non-cognitive abilities, performance, and skills that affect a person's ability to cope with environmental pressures and help him, or her overcome environmental stresses and needs [14, 15]. According to Mayer et al., emotional intelligence skills and competencies include four areas: the ability to perceive emotions, use emotions to speed thinking, understand emotions, and manage emotions [16]. Emotional intelligence was negatively correlated with anxiety [17], depression [18], and stress [17], and positively correlated with empathy [19], resilience [6], and academic performance [20].

Wilfredo Meza et al. 2022., studied on high school students in Peru, and believed that emotional intelligence is significantly correlated to success control of academic stress because student with higher emotional intelligence have better communication skills, self-control, perseverance, and self-motivation [21]. Kiran Narwal et al., 2018 examined the relationship between academic stress and emotional intelligence of visually disabled students. They suggested that emotional intelligence includes different skills that contribute to dealing with stress and successful performance in life. Yamin Chandra., 2020, analyzes the relationship between the perception of academic stress, coping strategies, and emotional intelligence among Indian students during the Covid-19 pandemic. They reported that students with high emotional intelligence show lower academic stress and positive coping style during a pandemic situation [22]. Earlier studies on college students in India [23], USA [24], Spain [6], China [25], and Iran [26], suggested emotional intelligence includes various skills that contribute to regular individuals’ ability to accept reality, solve emotional problems, have better levels of mental and physical health, and helps people to deal with stress and crisis. Therefore, it is seen that emotional intelligence could be a part of positive psychology [27].

Entering the university is a very sensitive event in the life of active and efficient forces in any country, which is accompanied by many changes in their personal and social relationships [28]. At the same time, medical university students are highly exposed to stressors related to community health due to the nature of their profession [29]. Although, several essential mechanisms related to adaptive habits and coping with stress dominate the possible vicissitudes that may occur, a high level of academic stress has been implicated as the main barrier to academic success and emotional problems. In Iran, a different survey reported that 81.1% of students had the stress of grades and tests [30], and 59.2% of university students reported that academic stress was unbearable. Therefore, academic stress and its mental and physical outcomes remain a serious public concern among students [14, 15]. According to this evidence, developing intervention-based novel strategies such as emotional intelligence skills can be effective to control stress and alter academic success according to students’ needs [27]. Despite the positive effect of emotional intelligence to control stress, few experimental studies have linked academic stress with students’ academic success. Likewise, the interaction between health education interventions and emotional intelligence skills has not been well evaluated [17, 27], and available data is still unclear on how/where students’ emotional intelligence can influence students ‘ability to deal with stress and crisis [27].

To address this gap, we conducted an educational intervention to explore the effect of emotional intelligence on medical students and their skills. The aims of our study were: (1) to evaluate the effect of emotional intelligence training on the level of emotional intelligence and academic stress among Iranian medical students, and (2) to measure the impact of such training on regulating and controlling students’ reactions to stressors. Our hypothesis was that effective training-based emotional intelligence skills (self-awareness, self-management, social awareness, and relationship management) could decrease students’ academic stress components including frustration, conflicts, pressures, self-imposed, changes, physiological, behavioral, emotional, and cognitive appraisal.

## Methods

### Study design and setting

This study consists of two parts: descriptive and quasi-experimental. In the first part, a descriptive study was performed on 200 students of Neyshabur university of medical sciences in the 2018–2019 academic year to determine the predictive power of emotional intelligence components. In order to determine the sample size based on Khodadadi et al.(2018) [31], study and considering the prevalence of stress equal to 12%, error level 0.05, accuracy 0.1 and the population size of 800 people, the sample size was determined based on the following formula about 200 people.

$$n=\frac{N{\mathcal{z}}^{2}p\left(1-p\right)}{N{d}^{2}+{\mathcal{z}}^{2}p\left(1-p\right)}$$

In the second stage, in order to perform training and intervention using the following formula and taking into account the first type error 0.05, power 80% of the ratio equal to the sample volume in the two groups (*r* = 1) and the expected effect size is 0.5, A total of 65 eligible students were studied for each group (130 students in total).

$$n=\left(\frac{1+r}{r}\right)\frac{{\left({z}_{1-\frac{\alpha }{2}}+{z}_{1-\beta }\right)}^{2}}{{d}^{2}}+\frac{{{z}_{1-\alpha /2}}^{2}}{2\left(1+r\right)}$$

**Fig. 1:**
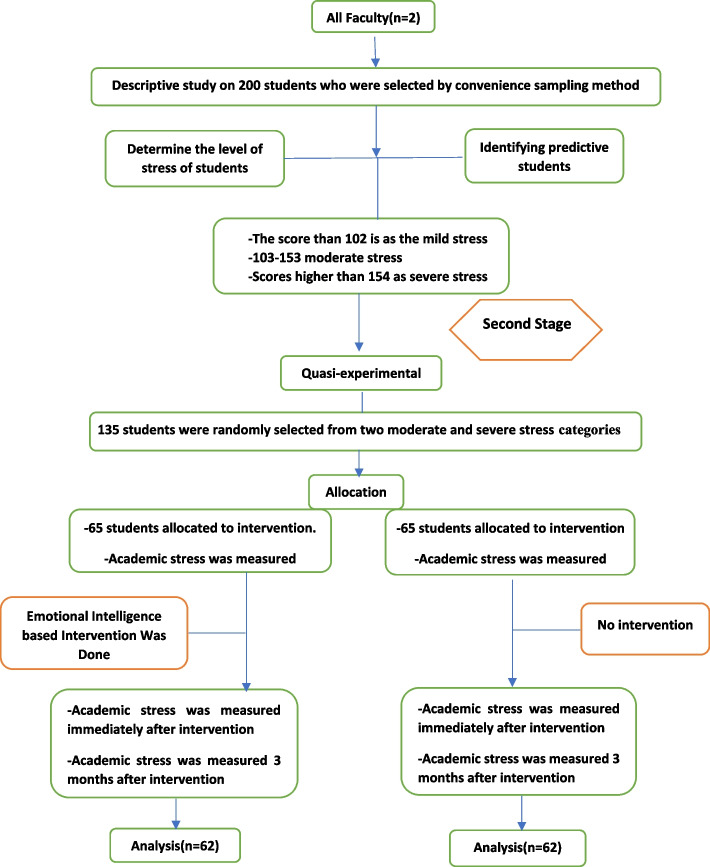
Flow of participants through each stage of the programme

The results of the descriptive study showed that stress was divided into three levels, the score lesser than 102 was considered mild stress, scores 103–153 were moderate stress and scores higher than 154 were considered severe stress. Then, 130 people with moderate to severe stress were randomly selected and divided into intervention and control groups (Fig. 1), and they completed the baseline (before intervention) and follow-up (post-intervention and 3 months after intervention) process.

### Characteristics of participants

Inclusion Criteria Studied at Neyshabur university of medical sciences and agreed to participate in the study. Exclusion criteria were: the declaration of non-cooperation during the study, absence of more than one session in training sessions, student withdrawal from the college for reasons such as dropping out of school or transfer and having an illness or mental problem or taking psychotic medication.

### Instruments

We used the Bradbury and Jane Graves emotional intelligence questionnaire, and the Gadzella academic stress questionnaire to examine students’ intelligence questionnaire and academic stress. Key elements of the study design were to examine how self-awareness, self-management, social awareness, and relationship management influence the level of academic stress components including environmental changes, self-imposed, physiological, and emotional problems, behavior, academic frustration, conflicts, and pressures among college students in Iran. Likewise, personal questionnaires were used to collect relevant data related to student demographic information (age, gender, field of study, academic years, grade, marital status, and residence status).

#### Bradbury and graves emotional intelligence inventory (EI)

Emotional intelligence (EI) was assessed by the Bradbury and Graves questionnaire consisting of 28 items and 4 dimensions as follows: self-awareness, self-management, social awareness, and relationship management. The self-management dimension contains 6 questions to assess conscientiousness, adaptability, trustworthiness, self-control, achievement drive, and initiative. Social Awareness involves 3 competencies to measure an individual’s service orientation, organizational awareness, and empathy.

Relationship management involves 8 questions to assess influence, conflict management, leadership, developing others, building bonds, leadership, change catalyst, communication, leadership, collaboration, and teamwork. Self-Awareness consists of 4 competencies to measure accurate self-assessment, self-confidence, and emotional self-awareness [32]. five-point Likert scale ranged from 1 (strongly disagree) to 5 (strongly agree). The total score ranged from 28 to 140, indicating high EI (81–140), normal EI (61–80), and low (60 > EI). Higher scores indicate higher emotional intelligence [33].

The validity and reliability of this questionnaire have been confirmed by Ganji et al. In Iran [32]. Their findings confirmed that this tool is the valid measure of EI among Iranian student because the ratio of the chi-square (× 2) statistic to its degree of freedom < 5, root mean square error of approximation (RMSEA) < 0.06, and standardized root mean residual (SRMR) < 0.05. Other indices such as the adjusted goodness-of-fit index (ANFI), the comparative fit index (CFI), and goodness of fit (GFI) were 0.97, 0.92, and 0.94, respectively. The reliability coefficients (Cronbach’s α) of self-awareness, self-management, social awareness and relationship management were 0·73, 0.87, 0.78, 0.76, and 0.82 respectively. Likewise, the total Cronbach’s α for this questionnaire was 0.92 indicating a strong internal consistency of this questionnaire [32].

#### Gadzella’s academic stress inventory

Gadzella’s Academic Stress Inventory was used to evaluate students’ academic stress based on 2 main categories and 51 items. The first category is divided into 5 subscales (frustration, conflicts, pressures, self-imposed, and changes) to measure students’ stress. Frustrations consist of 7 questions, evaluate student’s experiences about delays in achieving their goals, failure to reach set goals, socially being unacceptable, daily hassles, lack of sources, denials of opportunities, and, dating disappointments. Conflicts consist of 3 questions to examine one’s choices between two or more desirable or undesirable alternatives. Pressures include 4 questions to assess one’s deadlines, competitions, interpersonal relationships, and overload of activities. Self-imposed subscales consist of six items to evaluate one’s desire to compete, worries about everything, anxiety in test-taking, solutions to problems, to be loved by all, and procrastination. Changes in this tool include 3 items to assess numerous changes at one time, disruptive goals and life, and unpleasant experiences [34].

The second category is divided into 4 subscales (physiological, behavioral, emotional, and cognitive appraisal) to measure reactions to stressors. The physiological subscale includes 14 items that evaluate one’s experiences with trembling, exhaustion, sweating, rapid movements, stuttering, backaches, skin reactions, stomach problems, breathing problems, arthritic pains, headaches, and weight gains or weight losses. Behavioral subscales consist of 8 questions to assess one’s experiences with abuse of self or others, smoking excessively, using defense mechanisms, crying, attempting suicide, being irritable toward others, and separating oneself from others. Emotion includes 4 items to evaluate one’s experiences with anger, grief, fear, and guilt. Cognitive appraisals consist of 2 questions to assess whether appropriate strategies lead to solving stressful situations. Students' stress level is rated from 1 (mild) to 3 (severe) [33]. Then, a five-point Likert scale is used to rate 51 items from 1 (never student analyze and think the stressful situations) to 5 (most of the time student analyze and think the stressful situations). Higher scores indicate higher academic stress and more responses to stress [35].

The validity and reliability of the scale in Iran were confirmed by Shokri et al., [36]. Their findings confirmed that this tool is the acceptable measure of academic stress among Iranian students because × 2 statistic to its degree of freedom < 5, RMSEA < 0.06, and SRMR < 0.05. Other indices including ANFI, CFI, and GFI were 0.91, 0.98, and 0.92, respectively. Likewise, Cronbach’s α of frustration, conflicts, pressures, self-imposed, and changes were 0.74, 0.79, 0.70, 0.75, and 0.77, respectively. Likewise, Cronbach’s α of physiological, behavioral, emotional, and cognitive appraisal were 0.84, 0.80, 0.88, and 0.77. Likewise, the total Cronbach’s α for this questionnaire was 0.82 indicating a strong internal consistency for this questionnaire [34, 36].

### Intervention

This study was conducted from October 2020 to March 2020 based on two groups (intervention and control). For the intervention group, the medical student received a full training program and consulting support, while a student in the control group did not receive any training program. In the intervention group, the student received 2-month training that included six 2-h sessions (face to face) every 10 days were conducted. Likewise, internet messaging services and phone contact were used for counseling support. Educational training was conducted by a focus group, health educator, and clinical psychologist. Likewise, educational pamphlets, teach-back videos, and reminder cards were used to conduct an intervention.

The details of each session were as follows: first session) discussion about problem-solving was done. Students were then encouraged to particulate in their various issues and problems, especially in the field of education, and then to analyze different ways of solving the problem. There was also a discussion about realism, and the use of its techniques in reducing academic stress; second session) the strategy of using more adaptation to the surrounding conditions to deal with possible tensions was expressed and discussed. Then, the importance of emotional self-awareness is examined, and brainstorming is given on the causes and solutions for eliminating negative emotions such as stress and even moderately positive emotions.

There was also a discussion about self-respect and ways to achieve and maintain it; third session) use of self-fulfillment in controlling academic stress was discussed. Brainstorming and question-and-answer are used to discuss the necessity and benefits of independence and how to achieve it; the fourth session) describes strategies for coping with stress and tension and managing strong emotions. There was also talk about improving interpersonal relationships and empathy and its role in solving life problems; fifth session) discusses the skill of positive thinking and its role in managing life's problems and ways to improve them, and the sixth session) all content was summarized, and a review of the proposed strategies based on emotional intelligence to deal with academic stress was presented.

### Statistical analysis

After collecting data pertaining to pre-test, post-test (immediately after intervention) and follow-up stage (3 month after the intervention) in the experimental and control groups, the Kolmogorov–Smirnov test was used to determine the distribution of variables in terms of normalization. Then, using descriptive statistics, namely, frequency distribution of variables, mean and standard deviation, data were described for both experimental and control groups. Analytical statistics were used to compare the mean of variables in the two groups according to the distribution of variables.

#### Ethical considerations

The study conduction process began after obtaining the approval from the Deputy of research and obtaining permission from the Ethics Committee (IR. MUMS. REC. 1398. 294) and coordinating with the officials of Medical University of Neyshabur. At first, the purposes of the research project were explained to the subjects, and after obtaining informed consent, the students were provided with questionnaires and completed it as self-report. The right to abandon participation was considered for those who were unwilling to cooperate.

## Results

In this study, the sample included 79.24% female and 20.76% male participants. Likewise, 49.24% of the study sample were 20–24 years old, of whom 43.85% were second-year students, most of whom were single (68%) and non-native speakers (68.46%) (Table 1). There were no significant differences (*P* > 0·05) in the demographic measures (gender, age group, field of study, academic year, grade, marital status and residence status) between control and intervention groups.

**Table 1:** Frequency distribution of demographic variables

Variable		Experimental (*n 65*)		Control (*n 65*)		Total		*P*-value
		*n*	*%*	*n*	*%*	*n*	*%*	
Gender	Female	47	72.3	56	86.2	103	79.24	0.05*
	Man	18	27.7	9	13.8	27	20.76	
Age group	< 20	21	32.3	18	27.7	39	30	0.85**
	20–24	28	43.1	36	55.4	64	49.24	
	> 24	16	24.6	11	19.6	27	20.76	
Field of Study	Nursing	5	7.7	5	7.7	10	7.7	0.58***
	Anesthesia	4	6.2	3	4.6	7	5.4	
	Surgery room	6	9.2	10	15.4	16	12.3	
	Public health	9	13.8	6	9.2	15	11.53	
	Environmental Health	8	12.3	6	9.2	14	10.7	
	Health Professional	11	16.9	6	9.2	17	13.07	
	Medical urgency	8	12.3	6	9.2	14	10.7	
	Food Science and Technology	3	4.6	2	3.1	5	4	
	Health Information Technology	5	7.7	12	18.5	17	13.07	
	Midwifery	6	9.2	9	13.8	15	11.53	
Academic year	First year	8	12.3	9	13.8	17	13.07	0.27**
	Second year	25	38.5	32	49.2	57	43.85	
	Third year	26	40	18	27.7	44	33.85	
	Forth year	6	9.2	6	9.2	12	9.23	
Grade	Associate degree	3	4.6	2	3.1	5	4	0.65**
	Masters	62	95.4	63	69.9	125	96	
Marital status	Single	47	72.3	41	63.1	88	68	0.26*
	Married	18	27.7	24	36.9	42	32	
Residence status	Native	24	36.9	17	26.2	41	31.54	0.18*
	Non-native	41	63.1	48	73.8	89	68.46	

^*^Chi-square

**Mann whitney

*** Fisher's exact test

*n* Number of participants

Table 2 shows that the effective subscales of emotional intelligence on the total academic stress score are: self-management (*p *< 0.001), relationship management (*p* < 0.001), and self-awareness (*p* = 0.01). This model predicts 27.1% of students' academic stress (Table 2).

**Table 2:** Results of linear regression before intervention to determine the most important components of emotional intelligence

Dependent variable	Independent variable	Beta	*P*	R2
Academic stress	Self-management	-0.272	0.001 >	0.271
	Relationship management	-0.216	0.001 >	
	Self-awareness	-0.130	0.019	

Intergroup analysis using independent t-test on the mean scores of self-awareness, self-management, social awareness, relationship management, self-imposed stress, physiological response, emotional response, behavioral response and overall score of academic stress and Mann–Whitney test in the mean scores of conflict, Changes, stress, failure and cognitive response showed that at each stage of the study, there was no statistically significant difference between the experimental and control groups (*p* > 0.05). But in the case of social awareness, failure, emotional response and the score of academic stress in the stage immediately and 3 months after the intervention, this difference was significant (*p* < 0.05) and in the case of stress, self-imposed stress, physiological response and behavioral response. This difference was significant (*p* < 0.05) at 3 months post-intervention (Table 3).

**Table 3:** Comparison of mean and mean structures of emotional intelligence and stress before, immediately after the intervention and three months later in the experimental and control groups

Variable	Group	Before intervention		After intervention		Follow up stage (3 months after)		*P*-value*
		Mean	SD	Mean	SD	Mean	SD	
Self-awareness	Experimental	21.29	6.39	24.04	6.33	24.30	6.25	0.01
	Control	21.23	6.46	21.27	6.41	21.41	6.20	0.14
	*P*-value**	0.95		0.041		0.042		
Self-management	Experimental	26.76	7.93	29.95	7.60	29.40	7.76	0.02
	Control	26.43	8.09	26.49	7.92	26.46	8.01	0.52
	*P*-value**	0.81		0.035		0.044		
Social awareness	Experimental	17.87	4.31	19.89	4.25	19.70	4.17	0.03
	Control	17.64	4.12	17.56	4.17	17.61	4.19	0.40
	*P*-value**	0.75		0.002		0.005		
Relationship management	Experimental	22.55	7.53	24.24	7.60	24.40	7.74	0.04
	Control	22.30	6.25	22.49	6.05	22.50	6.19	0.09
	*P*-value**	0.84		0.048		0.026		
Conflict	Experimental	10.27	3.63	8.29	3.83	8.13	3.57	0.011
	Control	9.44	3.64	9.40	3.83	9.26	3.71	0.16
	*P*-value***	0.170		0.043		0.044		
Changes	Experimental	10.04	3.68	9.70	3.41	9.52	3.54	0.77
	Control	10.01	3.32	10.06	3.70	10.29	3.45	0.32
	*P*-value***	0.822		0.416		0.169		
Pressure	Experimental	13.38	11.90	11.90	3.63	11.09	3.73	0.025
	Control	13.23	13.30	13.30	3.84	13.43	3.70	0.22
	*P*-value***	0.929		0.054		0.049		
Failure	Experimental	19.73	18.46	18.46	3.15	18.38	3.10	0.05
	Control	20.53	20.81	20.81	3.60	20.72	3.69	0.23
	*P*-value***	0.141		< 0.001		< 0.001		
Self-imposed stress	Experimental	21.70	4.10	20.56	4.07	20.43	3.80	0.043
	Control	21.86	4.21	21.76	4.10	21.93	4.21	0.46
	*P*-value**	0.83		0.09		0.03		
Physiological response	Experimental	30.84	7.59	28.58	6.84	28.21	7.12	0.011
	Control	29.93	6.00	29.89	6.22	30.58	6.13	0.12
	*P*-value**	0.451		0.025		0.044		
Emotional response	Experimental	15.58	2.60	13.95	2.75	13.16	2.57	0.025
	Control	15.06	2.58	15.18	2.41	15.18	2.62	0.41
	*P*-value**	0.25		0.008		0.02		
Behavioral response	Experimental	19.49	5.62	17.84	5.64	17.63	5.15	0.040
	Control	19.32	5.75	19.47	5.76	19.58	5.83	0.20
	*P-*value**	0.866		0.031		0.045		
Cognitive response	Experimental	7.40	1.99	7.30	1.99	7.24	1.70	0.49
	Control	7.47	2.05	7.29	2.19	7.36	1.84	0.59
	*P*-value***	0.804		0.946		0.605		
Total academic stress	Experimental	148.47	15.92	137.63	15.83	24.40	7.74	0.04
	Control	146.89	13.25	147.20	13.75	22.50	6.19	0.09
	*P*-value**	0.539		0.001		< 0.001		

^*^Friedman

^**^T-test

^***^Mann whitney

The results of intra-group test using Friedman test showed that the difference between the mean of self-awareness, self-management, social awareness and relationship management in the immediate stage and 3 months after the intervention compared to before the intervention was significant only in the experimental group (*p* < 0.05). There was no significant difference in the control group (*p* > 0.05). But the results of intra-group test using Friedman test showed that the difference between the mean scores of changes in the immediate stage and 3 months after the intervention compared to before the intervention was significant only in the control group (*p* < 0.05). And the results of intra-group test on the difference between the mean scores of conflicts, stress, failure, self-imposed stress, emotional response, behavioral response, cognitive response shown in the stage immediately and 3 months after the intervention compared to before the intervention in none of the educational groups was not significant (*p* > 0.05). As to the difference between the mean score of physiological response and the overall score of academic stress, the results of intra-group test using Friedman test showed that the mean difference in the stage immediately and 3 months after the intervention compared to before the intervention in each of the two educational groups was significant (*p* < 0.05). (Table 3).

## Discussion

This study explores the effects of emotional intelligence training on the level of emotional intelligence and academic stress among medical students. These results supported our hypothesis, which showed that educational intervention based on emotional intelligence could significantly improve student’s emotional intelligence skills and decreased their academic stress and reactions to stressors. The results obtained in the present study showed that the overall level of emotional intelligence and the score of its dimensions (develop self-awareness, self-management, social awareness, and student relationship management) in the experimental group significantly exceeded those in the control group. This finding is in line with those of previous surveys [37, 38]. They confirmed the validity of total-trait emotional intelligence on increasing the amount of emotional intelligence in university students [37, 38]. In our study, the intervention involves developing student’s skills using real communication situations that student experience, which could improve student’s problem-solving skills, cultivate emotional regulation, and practice, and encourage them to share their feeling and use emotional skills. However, we suggest the stability of such intervention training needs to be evaluated in the future study.

This study showed that emotional intelligence training had a significant direct effect on regulating and controlling students’ reactions to stressors. Overall, the significant change in emotional intelligence scores in the experiment group may cause improved students’ physiological, behavioral, and emotional responses. This result is consistent with several studies that demonstrated that emotional intelligence training is effective to improve students’ and adolescents’ reactions to types of stressors [26, 39]. They indicated that individuals with higher levels of emotional intelligence cope better with the emotions or reactions related to stressful situations because they are able to accurately regulate and control their mood condition, know when and how to explain their feeling and sense, and can effectively appraise and perceive their emotions [39].

Our training was effective in decreasing students’ academic stress scores, and improving student’s frustration, conflicts, pressures, and self-imposed. It has seemed that the development of Emotional intelligence skills leads to improve students’ ability to manage and regulate their stress and enhances adaptive coping skills to decrease stress. Additionally, more studies have.

shown emotional intelligence skills, including self-management, relationship management, and self-awareness were significant mediators of percived stress among university students. Along with this study, Naik [40] obtained the most important factors of emotional intelligence: self-awareness and self-management. Goleman [41] considers emotional intelligence as an essential element for success in life and work in today's challenging social environment. He believes that emotional intelligence enables people to face the high pressures and severe challenges of social and perceptual development. Rakhshani et al. [15]. Likewise, the relationship between emotional intelligence and job stress was also examined, which was significant and inverse, showing that self-awareness, social awareness, and income predicted 25% of nurses' job stress. Of course, the tool used in the study by Rakhshani et al. different from the tools used in our research. What's more, the target groups and types of stress for the studies were different. Our study examined academic stress, but the study by Rakhshani et al. examined job stress.

Ruiz-Aranda et al. [42] found that participants with higher emotional intelligence reported lower levels of perceived stress. Which is in line with the study of Trigueros et al. [6] and our study. Emotional intelligence has a significant and inverse relationship with stress. In contrast, Sen et al. [43] in their study in India showed that there was no significant relationship between emotional intelligence and perceived stress, which is a difference in results from the tools used and the different target groups. Rostami et al. [44], shown in their study that there is a statistically significant difference between participants' emotional intelligence and stress coping styles. The results of the present study showed a significant inverse relationship between each of the components of emotional intelligence with academic stress coping skills. This item means that by increasing each of the components of self-awareness, self-management, social awareness and relationship management, academic stress decreases. Hence, by increasing each of these components, it is possible to reduce academic stress in students.

In a study aimed at identifying the causal relationship between emotional intelligence and academic stress by Yoo et al. [45], it was found that academic stress is indirectly affected by emotional intelligence. Findings from Frazier et al.'s [46] study showed that graduate students with higher emotional intelligence scores had less perceived stress. Therefore, increasing emotional intelligence skills can help reduce stress and create better stress coping skills. In a study, Miri et al. [47] examined the relationship between emotional intelligence and academic stress in students of Birjand university of medical sciences, which showed that there is no significant correlation between students' emotional intelligence and academic stress scores. The results of this study differ from ours. However, the tools used in this study are different from those used in our study. In addition, target group, cultural and geographical differences are also effective for differences in results.

Emami Moghadam, et al. [3] in their study, which aimed to determine the effect of emotional intelligence skills training program on high school girls 'academic stress, showed that the mean of students' academic stress scores after the intervention in the experimental group from 136 ± 23.7 to 126 ± 23.01 decreased while the mean scores in the control group did not change. This study is consistent with our study that the total score of academic stress in the experimental group after educational intervention decreased from 148.4 ± 15.9 to 136.8 ± 14.8, while the control group increased from 146.8 ± 13.2 to 148.3 ± 13.2. The average score of academic stress in our study was higher than the average of students' academic stress in the study by Emami Moghadam et al. the reason for this is the students' fields of study in our study that medical students suffer more stress than other fields due to the nature of their field. However, the results show that training in emotional intelligence skills has been able to significantly reduce the average stress in the experimental group while in the control group there has been an increase in the average stress. The results of the study by Shahni et al. [48] and Nooryan et al. [49] consistent with this study, which reported that teaching the components of emotional intelligence can reduce stress. Therefore, anything that increases emotional intelligence can help manage emotions, including stress. Emotional intelligence skills enable a person to overcome negative emotions and prevent stress [3].

Aghajani et al. [50] performed an intervention based on emotional intelligence skills training on academic stress of nursing students. The results of the study showed a significant difference in the mean score of total academic stress in the experimental group, which is consistent with our study. Furthermore, in the study by Aghajani et al., there were no significant differences in conflict, academic stress, changes, and self-imposed stress, which is consistent with our study. But in the study of Aghajani et al. [50], the results of the study showed a significant difference in the mean of emotional response and physiological response in the experimental group compared to the control group these results are different from our study, the reason may be that the Meyer and Salvia model was used as an educational model for the study by Aghajani et al. [50] another reason is the difference in outcomes during the follow-up period, in our study the questionnaire was completed 12 weeks after the educational intervention, but in the study by Aghajani et al. [50] it was done two weeks later. But finally, in two studies, teaching the components of emotional intelligence has been able to improve academic stress.

### Limitations

In this study, some limitations need to be noted. First, results were obtained based on self-report tools, which lead to the possible bias in results. Second, we could not identify causal relationships because this was a relational study: therefore, our results need interpret for each student’s perspective. Third, mediator items may not sensitive enough to detect all dimensions of emotional intelligence in natural setting or to reflect all changes during intervention and follow-up due to the multiple skills and strategies that were targeted in the training program. Additionally, in the 3 months after the intervention, due to the prevalence of Covid-19 virus and the closure of students, filling out the questionnaires was delayed, and we had to complete the questionnaires in absentia.

## Conclusion

The results indicated that the intervention program based on emotional intelligence skills can effectively improve students’ emotional intelligence skills and decreased their academic stress and reactions to stressors. It appears that the use of the components of emotional intelligence could offer a promising way to develop coping skills with academic stress. This has important implications for policy makers and researchers to include emotional intelligence cskills in the design and implementation of educational programs among medical students to control and reduce their academic stress and their ability to study without stress. Such a psychological intervention program is multiphased as it will help students, researchers, medical educators, and university administration to better understand the psychologicalstate of students. It is significant for medical educators that they can promote education quality by managing stress and higher emotion among their students. It would be a great help for university administration to develop stress coping strategies and emotional intelligence in students through promoting quality education for effective learning, which may be effective for a successful future. However, future research is still required to develop the sustain a positive training program to test emotional intelligence skills in a larger population whether such training is effective to regular and control the incidence of academic stress or is mediated by environmental and individual factors.

## Data Availability

Datasets used and/or analyzed during the current study are available from the corresponding author on reasonable request.

## Abbreviations

EI: Emotional intelligence
x^2^: Chi-square
RMSEA: Root Mean Square Error of Approximation
SRMR: Standardized Root Mean Residual
ANFI: Adjusted Goodness-of-fit Index
CFI: Comparative Fit Index
GFI: Goodness of Fit Index
